# Protocol for the MERINO study: A randomized placebo-controlled trial assessing the efficacy, safety, and cost-effectiveness of methotrexate in people with erosive hand osteoarthritis

**DOI:** 10.1016/j.ocarto.2024.100558

**Published:** 2024-12-15

**Authors:** Alexander Mathiessen, Line Gaundal, Joseph Sexton, Dag Sjølie, Pernille Steen Pettersen, Barbara Slatkowsky-Christensen, Ida Kristin Haugen

**Affiliations:** a Center for Treatment of Rheumatic and Musculoskeletal Diseases (REMEDY), Diakonhjemmet Hospital, Oslo, Norway; b Department of Radiology, Diakonhjemmet Hospital, Oslo, Norway

**Keywords:** Methotrexate, Erosive hand osteoarthritis, Pain, Function, Imaging

## Abstract

**Objective:**

Previous studies on the efficacy of methotrexate in people with hand osteoarthritis (OA) have shown conflicting results. The MERINO trial aims to investigate the efficacy and safety of methotrexate in people with painful inflammatory erosive hand OA.

**Design:**

In total 163 participants with erosive hand OA, synovitis by ultrasound, and finger joint pain of 40–80 ​mm on a visual analogue scale (VAS) will be recruited from a rheumatology outpatient clinic. Participants are randomized 1:1 to receive either encapsulated oral methotrexate 20 ​mg/week or placebo for 12 months in a double-blind manner. The primary endpoint is VAS finger joint pain at 6 months. Key secondary outcomes are hand function by the Australian/Canadian hand index (AUSCAN) at 6 months and radiographic progression by the Verbruggen-Veys anatomical phase scoring system at 12 months. Other secondary endpoints include hand stiffness, disease activity, health-related quality of life, grip strength, clinical joint counts, synovitis by ultrasound and MRI, bone marrow lesions by MRI, cost-effectiveness, and symptoms in knees and hips. Adverse events will be recorded. The primary analysis will be performed on full analysis set.

**Conclusions:**

The findings of this trial will be clinically relevant for patients with erosive hand OA and may influence future treatment recommendations.

**Clinical trial registration:**

EU CT number: 2023-510523-30-00, NCT04579848.

## 1 Introduction

Erosive hand osteoarthritis (OA) is a severe form of hand OA characterized by radiographic central erosions and a more aggressive clinical course, including more severe joint pain, stiffness, synovitis, and faster progression of structural damage compared to non-erosive OA [1]. In the general population of Framingham (USA), 10 ​% of women and 3 ​% of men above 40 years had erosive hand OA [2]. The limited treatment options for erosive hand OA (and OA in general) underscore the importance of developing more effective therapies for this disease.

Previous studies have shown that synovitis is associated with pain and future disease progression [[3], [4], [5], [6]], and people with erosive hand OA have more severe synovitis than people with non-erosive disease [1]. This provides a rationale for testing the anti-inflammatory drug methotrexate in the management of inflammatory erosive hand OA. The treatment recommendation for hand OA from the European Alliance of Associations for Rheumatology (EULAR) highlights the need for well-designed, larger randomized controlled trials (RCTs) to explore the efficacy of methotrexate [7].

In clinical practice, methotrexate is occasionally used off-label for OA patients with unmanageable pain and inflammation, although available treatment recommendations do not currently endorse this practice [[7], [8], [9]]. Previous studies, including two open-label studies and one RCT, have indicated a symptomatic effect of methotrexate in knee and hand OA [[10], [11], [12]]. However, two RCTs have been performed on people with hand OA with conflicting results [13,14], possibly due to differences in methotrexate doses and study populations. Therefore, additional well-designed studies on the efficacy and safety of methotrexate in hand OA are needed to provide clear treatment recommendations.

The hypothesis of the Methotrexate in ERosive INflammatory Osteoarthritis (MERINO) trial is that methotrexate will be effective in reducing pain and other symptoms, inflammation, and structural progression in people with erosive hand OA, with good safety and acceptable cost-effectiveness.

## 2 Material and Methods

### 2.1 Study design and setting

**Fig. 1:**
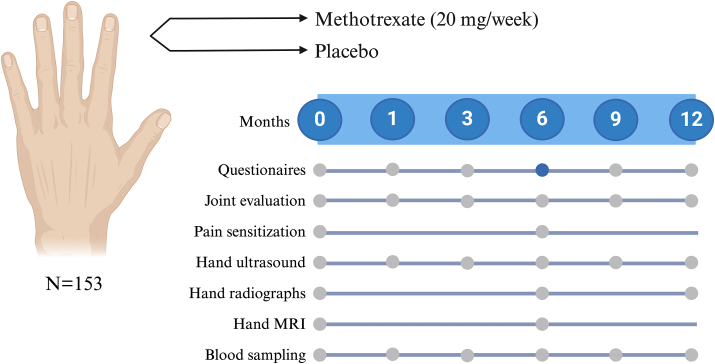
Overview of the study. Dark blue dot indicates timing of the primary endpoint. MRI ​= ​magnetic resonance imaging.

The MERINO trial is a 12-month, investigator-initiated, randomized, double-blinded, placebo-controlled, parallel-group, single-center, phase IV superiority study designed to assess the efficacy, safety, and cost-effectiveness of methotrexate in adults with painful erosive inflammatory hand OA. The overview of the study is illustrated in Fig. 1. The study involves six visits in addition to screening. The trial began recruitment in August 2021. We anticipate completing recruitment by 2025, with data collection concluding in 2026.

### 2.2 Study participants

The study population consists of Norwegian adults with symptomatic erosive inflammatory hand OA, who do not respond adequately to, are unable to tolerate, or have contraindications to paracetamol and/or non-steroidal anti-inflammatory drugs (NSAIDs). Details regarding the inclusion and exclusion criteria are shown in Table 1.

**Table 1:** Inclusion and exclusion criteria in the MERINO trial.

Inclusion criteria
• Finger joint pain 40–80 ​mm on 0–100 ​mm VAS with insufficient pain relief from, inability to tolerate or contra-indications to paracetamol and/or NSAIDs. • Hand symptoms (pain, aching, or stiffness) on most days (i.e. more than 50 ​% of the days) the previous 6 weeks. • Hand OA according to the American College of rheumatology (ACR) criteria. • At least one DIP or PIP joint in the 2nd-5th fingers with radiographic pre-erosive (J-phase) or erosive disease (E-phase) according to Verbruggen-Veys anatomical phase system).^a^ • At least two DIP or PIP joints with ultrasound-detected power Doppler signal of grade ≥1 or grey scale synovitis grade ≥2 on 0–3 scales.^a^
Exclusion criteria
• Contraindications to methotrexate: o Abnormal renal or liver function.^b^ o Lung fibrosis (maximum 6 months old x-ray), active infection, or reduced haematopoiesis (i.e. anaemia, leukopenia and/or thrombocytopenia). o Premenopausal women and fertile men not using contraceptives^c^; planned pregnancy within 18 months after screening (men/women), and pregnancy, breastfeeding or insufficient anti-conception therapy (women). o Alcohol or other drug abuse in the last year. o Intolerance to lactose. • Chronic inflammatory rheumatic diseases (such as RA, PsA or gout), active inflammatory bowel disease, or positive rheumatoid factor or anti-CCP antibodies. • Other severe co-morbidities.^d^ • Other likely causes of hand symptoms.^e^ • Oral or intra-muscular steroids in the previous month. • Intra-articular treatments or aspirations of any kind of hand joint the previous 3 months. • Analgesics or NSAIDs, unless stable dosage for ≥1 month. • SYSADOA, unless stable dose for ≥3 months.^f^ • DMOADs previous three months.^g^ • Scheduled hand surgery during the study. • Planning to start other treatment for hand OA during the study. • Not able to adhere to the study visit schedule and protocol requirements.

ACR=American College of Rheumatology; DIP ​= ​distal interphalangeal; anti-CCP ​= ​Anti–cyclic citrullinated peptide; DMOAD ​= ​Disease modifying osteoarthritis drugs; NSAIDs ​= ​non-steroidal anti-inflammatory drugs; PIP ​= ​proximal interphalangeal; PsA ​= ​psoriatic arthritis; RA ​= ​rheumatoid arthritis; SYSADOA=Symptomatic slow-acting drugs for OA; VAS ​= ​visual analogue scale.

a Ultrasound abnormalities and radiographic erosions do not have to be present in the same joint.

b Abnormal renal function defined as serum creatinine >142 ​μmol/L in women and >168 ​μmol/L in men, or a GFR <40 ​mL/min/1.73 ​m^2^. Abnormal liver function defined as transaminases above upper normal limit, active or previous hepatitis B or C infection, or known cirrhosis.

c Contraception should be maintained during treatment and until the end of systemic exposure, i.e. 3 months after methotrexate discontinuation. Sufficient anti-conception therapy consists of intra-uterine device (coil) or hormonal anti-conception (birth control pills, implant, intra-uterine system, dermal patch, vaginal ring, or injections).

d Such as hemochromatosis, fibromyalgia, psoriasis, blood dyscrasias and coagulation disorders, history of malignancy (except successfully treated squamous or basal cell skin carcinoma), uncontrolled diabetes mellitus, severe hypertension, unstable ischemic heart disease, severe heart failure, severe pulmonary disease, severe and/or opportunistic infections and/or chronic infections, active tuberculosis, positive human immunodeficiency virus status, recent stroke, bone marrow hypoplasia, or demyelinating diseases of central nervous system.

e Such as thoracic outlet syndrome, carpal tunnel disease, diabetic cheiropathy, injury in finger joints previous 6 months, or palmar tenosynovitis/trigger finger.

f Glucosamine sulfate, glucosamine hydrochloride, chondroitin sulfate, hyaluronic acid, avocado soybean unsaponifiables, and diacerein. Stable dose is required throughout the study.

g Existing or in pipeline, such as: strontium ranelate, sprifermin, Wnt pathway inhibitor, hydroxychloroquine, colchicine, tumor necrosis factor-inhibitors, intra-articular capsaicin, anti-nerve growth factor monoclonal antibodies, and Cathepsin K inhibitor.

### 2.3 Recruitment procedure

Patients with hand pain or suspected (erosive) OA at four outpatient clinics in the South-Eastern Health Region (Diakonhjemmet Hospital, Vestre Viken/Drammen Hospital, Martina Hansens Hospital, and Østfold Hospital Trust) are potential participants. Treating physicians identify eligible patients and inform the study team at Diakonhjemmet Hospital, who then contact these patients for information, consent, and screening. Potential participants are also identified based on erosive status on radiographs taken at Diakonhjemmet Hospital for other purposes, such as clinical studies, or through direct contact with the study team.

### 2.4 Patient and public involvement

Two patient research partners with hand OA have actively contributed during the planning phase, and assisted in designing the study, selecting assessments and questionnaires, and refining the informed consent form and participant information letters. Their input will continue throughout the execution of the trial and in the dissemination of its results.

### 2.5 Randomization, allocation concealment, and blinding

Participants are randomly assigned to either methotrexate or placebo in a 1:1 ratio using an allocation list based on permuted block randomization with random block sizes, generated by an independent statistician. Allocation to treatment groups follows a sequential approach based on the randomization list. Detailed information regarding block sizes and allocation is documented in a separate file inaccessible to enrollment and treatment assignment personnel.

**Fig. 2:**
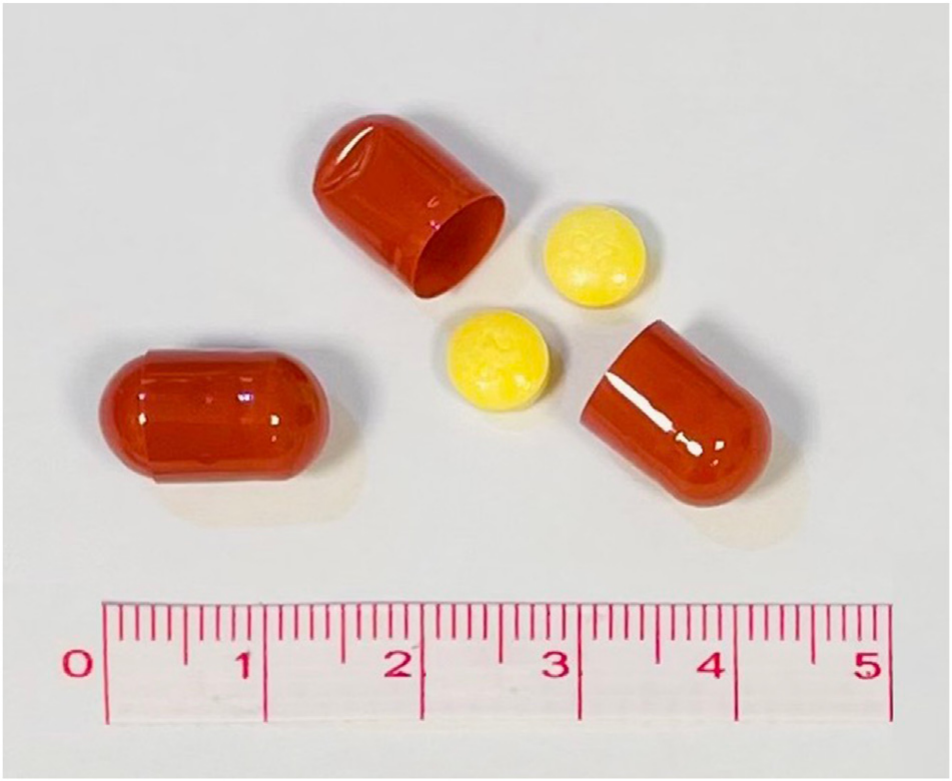
Capsules with methotrexate tablets.

To ensure blinding of participants and study personnel, both placebo and methotrexate are encapsulated (Capsugel® DBcaps® Over-Encapsulated Capsules, size B, colour Swedish orange, gelatin polymer) due to differences in appearance (Fig. 2). The encapsulation is done by Kragerø Tablet Production AS. The encapsulation will not affect the uptake of the drugs. The Medical Monitor may access the blinding code if necessary. Each participant receives a contact card for emergencies.

### 2.6 Interventions

Each capsule contains two tablets of 2.5 ​mg methotrexate (5 mg/capsule) or two tablets of placebo (Fig. 2). Participants are initially prescribed three capsules per week, providing a total of 15 ​mg methotrexate or placebo for the first two weeks. Subsequently, the dosage is increased to four capsules per week, totaling 20 ​mg methotrexate or placebo for the remainder of the study. In cases of intolerance, such as nausea, the dose can be reduced to three or two capsules per week. All participants also receive daily folic acid (1 ​mg) to mitigate anticipated adverse reactions to methotrexate [15].

Participants maintain a weekly diary to record their prescribed dosage regimen at home. Compliance is monitored and documented at each study visit. Before disposing their pillboxes, a study nurse conducts a pill count to track the number of study tablets dispensed and taken by each participant.

### 2.7 Concomitant medication

All concomitant medications are documented throughout the study. Participants can maintain a stable dosage of chondroitin sulfate, glucosamine, bisphosphonate, tetracycline, and estrogens. Analgesics (e.g., paracetamol) and NSAIDs (e.g., ibuprofen, diclofenac, etoricoxib, naproxen) are used as needed and recorded in a personal diary. Participants are advised to avoid using these medications within 48 ​h before a study visit or while completing questionnaires. The use of analgesics and NSAIDs is summarized and documented at each study visit. The study prohibits the use of systemic corticosteroids (intramuscular, intravenous and oral), corticosteroid injections in hand joints or around hand tendons, hyaluronic acid, or topical capsaicin during the trial.

### 2.8 Safety

Throughout the study, adverse events are systematically recorded, with any serious events promptly reported to the chief investigators within 24 ​h of acknowledgment. Treatment safety is evaluated following monitoring guidelines. As per recommendations from the Norwegian Rheumatology Association, participants undergo comprehensive blood tests, including full blood count, liver and kidney function, and electrolyte level measurements. These tests are conducted at screening, and subsequently at 1, 2, and 3 months, and then every 3 months. For fertile women, serum human chorionic gonadotropin levels are monitored at each visit throughout the treatment period and 3 months thereafter.

The investigator must follow up on significant adverse events until they recover, resolve, do not recover, have unresolved outcomes, recover with sequels, are fatal, or have an unknown outcome.

### 2.9 Endpoints

The primary endpoint is finger joint pain previous 48 ​h on a visual analogue scale (VAS; 0–100 ​mm) at 6 months (M06) of treatment. The key secondary endpoints are physical function according to the AUSCAN physical function subscale at M06 and radiographic damage according to the Verbruggen-Veys anatomical phase scoring system at M12. Several other secondary endpoints will be measured, as shown in Table 2.

**Table 2:** Overview of measurements.

Measurements	Time point (months)^a^
**Primary outcome**	
• Finger joint pain previous 48 ​h (VAS)	• 0, 6
**Secondary outcomes**	
• Finger joint pain previous 48 ​h (VAS)	• 0, 1, 3, 9, 12
• Thumb base joint pain previous 48 ​h (VAS)	• 0, 1, 3, 6, 9, 12
• Pain most painful finger joint previous 48 ​h (VAS)	• 0, 1, 3, 6, 9, 12
• Patient-reported hand disease activity previous 48 ​h (VAS)	• 0, 1, 3, 6, 9, 12
• Patient-reported pain in all body joints previous 48 ​h (VAS)	• 0, 1, 3, 6, 9, 12
• Pain in hand joints previous 48 ​h (hand figure)	• 0, 6
• Pain in other joints previous 48 ​h (body figure)	• 0, 6
• AUSCAN hand pain, stiffness and function	• 0, 1, 3, 6, 9, 12
• OMERACT/OARSI responder criteria	• 0, 1, 3, 6, 9, 12
• EuroQol – 5 dimensions – 5 levels	• 0, 1, 3, 6, 9, 12
• MHOQ pain, function, activities of daily living and work	• 0, 6
• Grip strength	• 0, 6
• Hip symptom, function, and quality of life (HOOS-12)	• 0, 6
• Knee symptom, function, and quality of life (KOOS-12)	• 0, 6
• Use of analgesics and NSAIDs	• 0, 1, 3, 6, 9, 12
• Tender and swollen hand joint counts	• 0, 1, 3, 6, 9, 12
• Physician-reported disease activity	• 0, 1, 3, 6, 9, 12
• Bilateral hand radiographs	• 0, 6, 12
• MRI of dominant hand	• 0, 6
• Ultrasound of hand joints	• 0, 1, 3, 6, 9, 12
• Ultrasound of knees and hips	• 0, 6
• Adverse events and safety	• 0, 1, 2, 3, 6, 9, 12
• Health economics^b^	• 0, 1, 3, 6, 9, 12
**Tertiary/exploratory outcomes**	
• Question about blinding • Hospital Anxiety and Depression scale • Pain Catastrophizing • Pain Sensitivity questionnaire • Patient acceptable symptom State (anchor question) • Minimal clinically important Improvement (anchor question) • Self-efficacy (ASES) • Pain sensitization • Soluble biomarkers • Omics analysis of whole blood	• 12 • 0, 6 • 0, 6 • 0, 6 • 0, 6 • 0, 6 • 0, 6 • 0, 6 • 0, 6, 12 • 0, 6, 12
**Demographics and clinical characteristics**	
• Demographics, lifestyle factors, hormonal factors (women), previous treatments, alternative treatments, preferred treatment, pain in hand joints previous 6 weeks (hand figure), ACR classification criteria, Comorbidity index, Symptom severity score and Widespread pain index	• 0
• Height and weight	• 0, 12
• Medication list	• 0, 1, 3, 6, 9, 12
• Blood pressure and heart rate	• 0, 1, 3, 6, 9, 12

ACR = American College of Rheumatology; AUSCAN = Australian/Canadian hand index; HOOS = Hip disability and Osteoarthritis Outcome Questionnaire; KOOS = Knee injury and Osteoarthritis Outcome Score; MHOQ ​= ​Michigan Hand Outcome Questionnaire; OARSI = Osteoarthritis Research Society International; OMERACT = Outcome Measures in Rheumatology; VAS ​= ​visual analogue scale.

a Month 0 includes both assessments at screening and at baseline.

b Health economics: A cost-utility analysis where direct and indirect healthcare costs are calculated, will be performed. We will collect data about intervention-related resource use, healthcare service use, and labor market status. The EuroQol 5 dimensions 5 levels (EQ-5D-5L) questionnaire will be used to collect descriptions of the participants´ health-related quality of life.

#### 2.9.1 Questionnaires

Participants complete self-reported questionnaires at the hospital visits scheduled at baseline (M00), month 1 (M01), month 3 (M03), month 6 (M06), month 9 (M09), and month 12 (M12) (Table 2). Most participants answer the questionnaires electronically in a web-based electronic case report form software (Viedoc™, Uppsala, Sweden). The questionnaires are also available in paper form if needed.

#### 2.9.2 Joint examination

A trained rheumatologist conducts joint examinations as outlined in Table 2. The clinical criteria for hand, hip, and knee OA specified by the American College of Rheumatology (ACR) are assessed [[16], [17], [18]]. Each knee/hip is assessed separately, and OA classification requires fulfillment of criteria in at least one joint. Bilateral hand joints are evaluated as a whole.

The examination includes assessment of soft tissue swelling, bony enlargement, and joint tenderness (absent/present) following guidelines from the EULAR handbook [19]. The bilateral 2nd-5th distal interphalangeal (DIP), 2nd-5th proximal interphalangeal (PIP), thumb interphalangeal (IP-1), 1st-5th metacarpophalangeal (MCP) and thumb base joints are examined. Global hand OA disease activity is evaluated on a visual analogue scale (VAS) (0–100 ​mm) by a physician.

Grip strength is measured three times in each hand using a Jamar dynamometer, with 15 ​s of rest between measurements [20]. The mean value of the three measurements is calculated.

#### 2.9.3 Quantitative sensory testing (QST)

The QST protocol is performed by trained study nurses. The protocol includes evaluation of 1) temporal summation, using punctate probes (MRC Systems GmbH) with exerted forces at the left distal radioulnar joint, then 2) pressure pain thresholds (PPT), using a hand-held digital algometer (Wagner FPIX25 or FPIX50) at a painful DIP or PIP joint and left part of trapezius muscle, and finally 3) conditioned pain modulation, using the blood pressure ischemic test while performing the post-CPM PPT test at the trapezius muscle. The temporal summation test is performed twice with 2 ​min rest between the two examinations, while the PPT is tested three times at each site with short intervals (approximately 30 ​s). The PPT at the painful finger joint is tested at the same joint at both M00 and M06. Detailed descriptions of the QST protocol can be found in previous publications [21,22].

#### 2.9.4 Imaging

Bilateral frontal hand radiographs (posteroanterior view) are obtained at screening, M06 and M12 (source to image-receptor distance: 115 ​cm; exposure: 46 ​kVp and 2 ​mAs). A trained reader evaluates the DIP, PIP, IP-1, MCP, first carpometacarpal (CMC-1) and scaphotrapeziotrapezoidal (STT) joint using a modified version of the Kellgren-Lawrence (KL) scale and the OARSI atlas [2,[23], [24], [25]]. The DIP and PIP joints are also scored according to the Verbruggen-Veys scoring system [26]. Additionally, a chest radiograph (front and side view) is performed as part of screening before initiating intervention and interpreted by radiologists at Diakonhjemmet Hospital.

Ultrasound of fingers, hips, and knees is performed using a General Electric logic S8 machine (GE, Medical Systems, Milwaukee, Wisconsin, USA) with a 6–15 ​MHz linear array transducer by an experienced rheumatologist. Participants will be seated opposite to the sonographer with their hands, wrists and forearms in prone position resting on a table. The bilataral DIP, PIP, IP-1, MCP, CMC-1 and STT joints are examined at all visits. The hand joints are scanned longitudinally from the radial to the ulnar side, and transverse scanning is performed in case of uncertainty. A preliminary ultrasound scoring system is used for assessment of grey scale synovitis (i.e. synovial hypertrophy and/or effusion) and power Doppler signals [27]. Grey scale synovitis is scored on a 0–3 scale, where 0 ​= ​normal, 1 ​= ​minimal, 2 ​= ​moderate, and 3 ​= ​severe pathology relative to the maximal volume of synovial hypertrophy and effusion. Power doppler signals need to be detected within synovial hyperthrophy and are scored on a 0–3 scale, where 0 ​= ​no flow in the synovium, 1 ​= ​minor, or single vessel signals (one or more), 2 ​= ​moderate, and 3 ​= ​major.

Bilateral hips are examined at M00 and M06 with the participant in a supine position and hips fully extended on the examination bed. Osteophytes on the femoral neck are scored on a 0–3 scale (0 ​= ​no, 1 ​= ​small, 2 ​= ​medium, 3 ​= ​large osteophytes), and the capsule is assessed as convex, flat, or concave.

Bilateral knees are examined at M00 and M06 with the participant in a supine position and the knees in extended position. The maximum score of grey-scale synovitis at the suprapatellar or parapatellar recesses in each knee is assessed on a 0–3 scale [28]. Osteophytes are assessed at the medial and lateral bone margins of the tibiofemoral joint, and scored on 0–3 scales in each compartment (0 ​= ​no, 1 ​= ​small, 2 ​= ​medium, 3 ​= ​large osteophytes).

MRI of the dominant hand (Siemens Aera 1.5T whole-body MRI scanner) is performed at M00 and M06 using a dedicated hand coil (Table 3). Intravenous gadolinium contrast (Clariscan 0.5 ​mmol/mL, 0.2 ​mL/kg body weight) is given unless contraindications (e.g. previous allergic reactions or glomerular filtration rate <40 ​mL/min). The MRIs are scored by a trained reader for synovitis and bone marrow lesions according to validated scoring systems [29,30].

**Table 3:** Details of MRI sequences.

	Coronal PD Dixon	Axial PD Dixon TSE	Coronal T1 VIBE WEPre-contrast	Coronal T1 VIBE WEPost-contrast
Echo time (ms)	42	36	6.91	6.91
Repetition time (ms)	2580	2390	16.4	16.4
Slice thickness (mm)	2	3.2	0.3i	0.3i
Spacing (mm)	0.6	0.6	0	0
Matrix (pixel size in mm)	528i x 768i (0.29i x 0.29i)	384i x 256i (0.3i x 0.3i)	896i x 628i (0.26i x 0.26i)	896i x 628i (0.26i x 0.26i)
FOV (mm)	220 ​× ​151.3	120 ​× ​80	230 ​× ​161.2	230 ​× ​161.2
Time (min:sec)	2:39	2:37	3:16	3:16

FOV ​= ​field of view; i ​= ​interpolated; mm ​= ​millimeters; ms ​= ​milliseconds; PD ​= ​proton density; TSE ​= ​turbo spin echo; VIBE ​= ​volumetric interpolated breath-hold examination; WE ​= ​water excitation.

### 2.10 Statistical analyses

Data will be analyzed primarily on the full analysis set population and repeated for the per-protocol population. A superiority test will be performed to assess the hypothesis of a between-group difference in VAS finger joint pain at M06. A p-value <0.05 will be considered statistically significant.

Key secondary endpoints including AUSCAN physical function at M06 and radiographic damage by Verbruggen-Veys anatomical phase scoring system at M12 will be tested hierarchically in this order and thus controlling for multiplicty, if the primary null hypothesis is rejected. The remaining secondary endpoints will not be adjusted for multiplicity.

Continuous secondary variables will be analyzed with linear regression models. Binary response variables will be analyzed using logistic regression models. The details of the statistical analyses will be presented in the statistical analysis plan. No interim analyses are planned for this trial.

Sample size is based on the difference in VAS finger joint pain (primary outcome). In previous hand OA trials, the difference between groups in VAS finger joint pain have ranged between 8 and 16 ​mm, with VAS pain of 42–55 for placebo groups and 33–42 for treatment groups (SD: 21–28 ​mm) [14,[31], [32], [33], [34], [35]]. To detect a between-group difference of 10 points with 80 ​% power in a one-sided ANCOVA-based test, we first required 74 participants in each group. During the study, the results from the METHODS trial allowed us to reduce our sample size to 65 participants in each group. From this data, we estimated the correlation between baseline and 6-months VAS pain to be 0.35, with a pooled SD of 17 and 24 for baseline and 6-months scores, respectively [13]. From this we concluded that 163 participants would be needed to satisfy our power requirement, accounting for a 20 ​% loss to follow-up.

### 2.11 Ethics and dissemination

Before trial inclusion, informed consent is obtained from all participants. At any point, participants can exit the trial without needing to justify their decision. Documents linking the participant identification numbers in the eCRF to their names are securely stored in locked cabinets. Paper-based data are secured in locked cabinets that have restricted access. All data will be de-identified.

The MERINO trial is officially listed on https://clinicaltrials.gov (NCT04579848). Approval for this study has been granted by the Norwegian Regional Committee for Medical and 10.13039/100005622Health Research Ethics (2020/187524) as well as the Norwegian Medicines Agency via the clinical trials information system (EU CT number: 2023-510523-30-00). The data protection officer at Diakonhjemmet Hospital has endorsed the trial. The trial adheres to the CONSORT guidelines for reporting randomized trials and all procedures adhere to the principles of the Helsinki Declaration.

Trial results will be published in international peer-reviewed journals. Furthermore, results will be reported to the Clinical trials information system within one year following the study's conclusion and will be shared with the participants of the study. Results will also be disseminated through peer-reviewed journals, conferences, patient organizations, and media channels.

## 3 Discussion

The MERINO study will be the first study on the efficacy and safety of methotrexate 20 ​mg/week in people with painful inflammatory erosive hand OA with data on symptoms, synovitis by imaging and clinical examination, and radiographic progression.

The MERINO trial will complement previous studies on the efficacy of methotrexate in hand OA, which have shown conflicting results. The METHODS trial included 97 participants with inflammatory hand OA and found a modest but potentially clinically meaningful reduction in pain over a 6-month treatment period with methotrexate 20 ​mg/week. In the METHODs trial [13], no data were available on imaging or laboratory outcomes, which is a clear benefit of the MERINO trial. Another RCT of 64 persons with erosive hand OA showed a positive trend with larger pain relief in patients treated with methotrexate versus placebo after 3 months, but the difference between groups was not statistically significant. However, the lack of inflammation in many patients and the low dose of methotrexate (10 ​mg/week) made it challenging to draw firm conclusions. Interestingly, fewer patients developed new erosions in the methotrexate versus placebo arm, suggesting a disease-modifying effect [14].

In the MERINO trial, we have chosen to have our primary endpoint on pain after 6 months, due to the slow onset of action by methotrexate. We have chosen 6 months rather than 12 months due to risk of loss to follow-up and reduced compliance towards the end of the study. Nevertheless, the treatment duration of 12 months allows for evaluation of prolonged effects on symptoms and structural progression. Since methotrexate is a systemic treatment with potential effects also in joints other than the hand, we also evaluate knee and hip symptoms and imaging findings.

Our goal is to advance the management of hand OA. We anticipate that our findings will hold significant clinical implications and influence future treatment recommendations for hand OA.

## Author contributions

All co-authors had substantial contribution to the conception or design of the work, or the acquisition, analysis or interpretation of data. AM, LG and IKH drafted the paper. All co-authors revised it critically for important intellectual content and gave their final approval of the version published. AM and IKH take the responsibility for the integrity of the work as a whole, from inception to finished article

## Role of the funding source

The MERINO trial is funded by South-Eastern Norway Regional Health Authority, Anders Jahre's Foundation for the Promotion of Science, Grete Harbitz's Foundation, and Dr. Trygve Gythfeldt og Wife's Research Foundation. The REMEDY center was funded by The Research Council of Norway (project number: 328657) and the Thon Foundation. The funders were not involved in the study design, data collection, writing the manuscript or the decision to submit the manuscript.

## Declaration of competing interest

**IKH** reports consulting fees from Novartis, GSK and Grünenthal, and speaker honorarium from Abbvie, outside of the submitted work. Other co-authors report no conflicts of interest.
